# Spatial Organization and Correlations of Cell Nuclei in Brain Tumors

**DOI:** 10.1371/journal.pone.0027323

**Published:** 2011-11-16

**Authors:** Yang Jiao, Hal Berman, Tim-Rasmus Kiehl, Salvatore Torquato

**Affiliations:** 1 Physical Science in Oncology Center, Princeton Institute for the Science and Technology of Materials, Princeton University, Princeton, New Jersey, United States of America; 2 Department of Laboratory Medicine and Pathobiology, Campbell Family Institute for Cancer Research, University of Toronto, Toronto, Ontario, Canada; 3 Department of Pathology, University Health Network, Toronto Department of Laboratory Medicine and Pathobiology, University of Toronto, Toronto, Ontario, Canada; 4 Department of Chemistry and Physics, Program in Applied and Computational Mathematics, Princeton Center for Theoretical Science, Physical Science in Oncology Center, Princeton Institute for the Science and Technology of Materials, Princeton University, Princeton, New Jersey, United States of America; University of Pennsylvania School of Medicine, United States of America

## Abstract

Accepting the hypothesis that cancers are self-organizing, opportunistic systems, it is crucial to understand the collective behavior of cancer cells in their tumorous heterogeneous environment. In the present paper, we ask the following basic question: Is this self-organization of tumor evolution reflected in the manner in which malignant cells are spatially distributed in their heterogeneous environment? We employ a variety of nontrivial statistical microstructural descriptors that arise in the theory of heterogeneous media to characterize the spatial distributions of the nuclei of both benign brain white matter cells and brain glioma cells as obtained from histological images. These descriptors, which include the pair correlation function, structure factor and various nearest neighbor functions, quantify how pairs of cell nuclei are correlated in space in various ways. We map the centroids of the cell nuclei into point distributions to show that while commonly used local spatial statistics (e.g., cell areas and number of neighboring cells) cannot clearly distinguish spatial correlations in distributions of normal and abnormal cell nuclei, their salient structural features are captured very well by the aforementioned microstructural descriptors. We show that the tumorous cells pack more densely than normal cells and exhibit stronger effective repulsions between any pair of cells. Moreover, we demonstrate that brain gliomas are organized in a collective way rather than randomly on intermediate and large length scales. The existence of nontrivial spatial correlations between the abnormal cells strongly supports the view that cancer is not an unorganized collection of malignant cells but rather a complex emergent integrated system.

## Introduction

Cancer is a highly complex and heterogeneous set of diseases. Heterogeneity occurs on a variety of length scales, including the genomic, phenotypic, cellular, tissue and metastatic intra-organ levels [1]–[5]. The rapid growth and resilience of tumors as well as the reproducible diagnostic classification of tumors based upon morphologic patterns make it difficult to believe that they behave as random, disorganized and diffuse cell masses and suggests instead that they are self-organizing, opportunistic systems [2], [3]. It is reasonable to expect that this self-organization would be reflected in the manner in which malignant cells are spatially distributed in their heterogeneous environment. Indeed, Thomlinson and Gray showed that in well-vascularized tumor environment, the malignant cells are often organized around blood vessels into “solid rods” (i.e., Krogh cylinders) with predictable cellular changes in the perivascular space [6]. In fact, one does not need to know the microvascular anatomy *a priori*; such information is reflected in the spatial arrangement of the cells. In addition, it is difficult to obtain information of how cells are spatially correlated on large length scales beyond the characteristic scale associated with a single Krogh cylinder. A crucial question then is how to systematically probe and extract the structural information in model-independent manner. It has been suggested recently [7] that the powerful theoretical machinery of heterogenous materials, developed in the physical and mathematical sciences [8], be brought to bear to characterize the structure and bulk properties of the heterogeneous tumor environment. In this paper, we employ techniques from the theory of heterogeneous media to characterize spatially optical images of the distribution of the nuclei of both benign brain white matter cells and brain glioma cells.

A spatial distribution of cell nuclei can be modeled as a distribution of points by identifying the geometrical centroids of the nuclei. Point distributions are one of the most popular and widely used models for many-particle systems in various branches of modern science, including condensed matter physics and materials science [8]–[10], statistical mechanics [11], discrete geometry [12], cosmology [13] and biology [8], [14]. It is of great interest to investigate how the points are spatially correlated with one another on small, intermediate and large length scales, since such information can reveal underlying mechanisms of the formation of a point distribition. The degree of spatial correlations among the points can vary from perfect long-range order (which occurs in a crystal structure [9]) to the absence of any spatial correlations. In particular, a completely uncorrelated point distribution, i.e., a Poisson distribution [8], can be obtained by randomly placing a large number of points in some domain (see Supporting Information S1 and supporting figure Fig. S1 with the associated legend). Thus, deviations of spatial statistics of a point distribution from those of the Poisson distribution provide a measure of the degree of spatial correlations. We employ Poisson point distributions as a reference system to characterize spatial correlations in the distributions of cell nuclei.

Local spatial statistics, such as the number of neighboring cells and cell areas are commonly used to characterize cell aggregates [8], [15], [16]. A systematic way of obtaining such statistics is to construct the Voronoi tessellation associated with the distribution of the cells. (A Voronoi tessellation is a subdivision of the plane into polygons, see the Results section for a precise definition.) Although the statistics of Voronoi polygon areas and number of neighbors can provide useful structural information for certain systems, such as epithelia [15], we find that they are not able to capture well the salient features of the spatial correlations in distributions of the nuclei of benign brain white matter cells and brain glioma cells nor clearly distinguish between the two. This motivates us to look for more sensitive microstructural descriptors to characterize spatial distributions of cell nuclei in normal and tumorous environments.

Specifically, we introduce a class of nontrivial statistical microstructural descriptors that arise in the theory of heterogeneous media [8] and employ them to characterize the spatial distributions of cell nuclei. These descriptors, which include the pair correlation function, structure factor and various nearest neighbor functions (defined in Materials and Methods), quantify how pairs of cell nuclei are correlated and distributed in space, e.g., how the position of a cell is influenced by another cell at a prescribed distance away. To the best of our knowledge, this is the first time that such spatial statistics have been applied in the analysis of histological images. By comparing the statistics of the nuclei distributions to the corresponding Poisson-distribution reference systems and by directly comparing appropriately scaled distributions of normal and abnormal cell nuclei (i.e., cells in a viable brain glioma environment), we show that their salient structural features are captured very well by the aforementioned correlation functions. In particular, we find that the abnormal cells pack more densely than normal cells and possess stronger short-range correlations. Moreover, we demonstrate that the distributions of abnormal cell nuclei possess nontrivial long-range spatial correlations, which appears to be a new and biologically significant observation. Our discovery of nontrivial spatial correlations between the abnormal cells on both small and large length scales strongly support the view that cancer is not a random collection of malignant cells but a complex emergent integrated system.

## Results

### Histological Images

Pixelized RGB color images were generated from sections of viable regions of glioblastomas in 13 individuals and from sections of brain white matter without significant pathologic abnormality in 20 individuals. From each glioblastoma, three images were obtained within areas tumor scored as 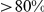 viable tumor cellularity as determined by our study pathologist (HB). From each individual without significant pathologic abnormality, three images were obtained at three different locations randomly selected. The size of each image is 1310 microns by 983 microns. Each image of benign brain white matter contains approximately 1700 cell nuclei, while each image of brain glioma contains approximately 5000 cell nuclei. Thus, cells in the tumor environment (i.e., abnormal cells) “pack” much more densely than normal cells with a number density 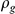 (i.e., number of cell nuclei per unit area) approximately three times larger than that of normal cells 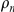, i.e., 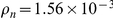 micron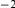 and 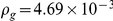 micron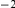. The 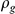 value is consistent with recently reported values for grade 3 and 4 glioma [17]. Note that 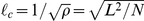 gives a characteristic length scale associated with a single cell, which is referred to as *characteristic neighbor distance*. Here we have 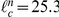 microns for normal cells and 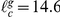 microns for cells in tumor environment.

### Mapping Distributions of Cell Nuclei to Point Distributions

**Figure 1:**
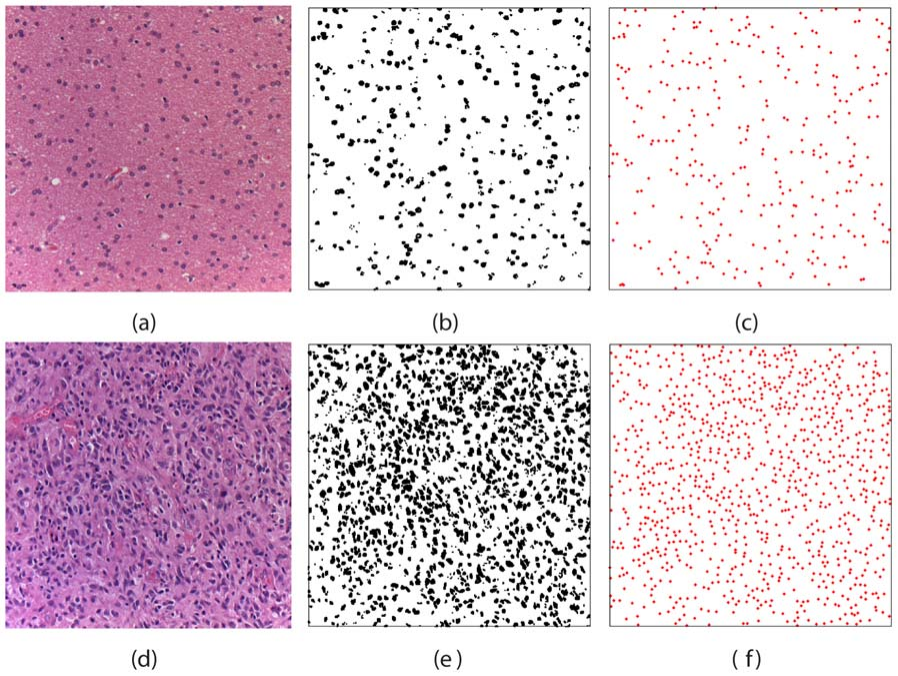
Distributions of nuclei of benign brain white matter cells (upper panels) and nuclei of brain glioma cells (lower panels).

The original color images are converted to gray scale images using MATLAB. Then a threshold value of grayness is chosen to make binary images such that the nuclei are shown as black clusters (see Fig. 1). The centers of the nuclei are then obtained by averaging the positions of pixels of their associated clusters. In this way, we map the distributions of cell nuclei into point distributions. Note that we have excluded histologically apparent non-tumoral structural heterogeneities such as blood vessels when thresholding the gray scale images. This allows us to focus on the spatial correlations of cell nuclei alone, which in fact contain information about such heterogeneities. For example, close to a blood vessel, the local number of density of cell nuclei is slightly higher. In addition, periodic boundary conditions are used for each point distribution (e.g., it is surrounded by periodic images of itself *ad infinitum*) in order to obtain the spatial statistics (i.e., the Voronoi statistics and correlation functions). The statistics for normal and abnormal cell nuclei are averaged over 60 and 39 individual images, respectively, to reduce noise and enhance common characteristics. Because the distributions of normal and abnormal cell nuclei do not possess the same number density, their structural statistics can not be directly compared to each other. Therefore, Poisson-point distributions at appropriate number densities are used as reference systems for comparison. Furthermore, the distributions are scaled to the same number density when the statistics are directly compared.

### Voronoi Statistics

**Figure 2:**
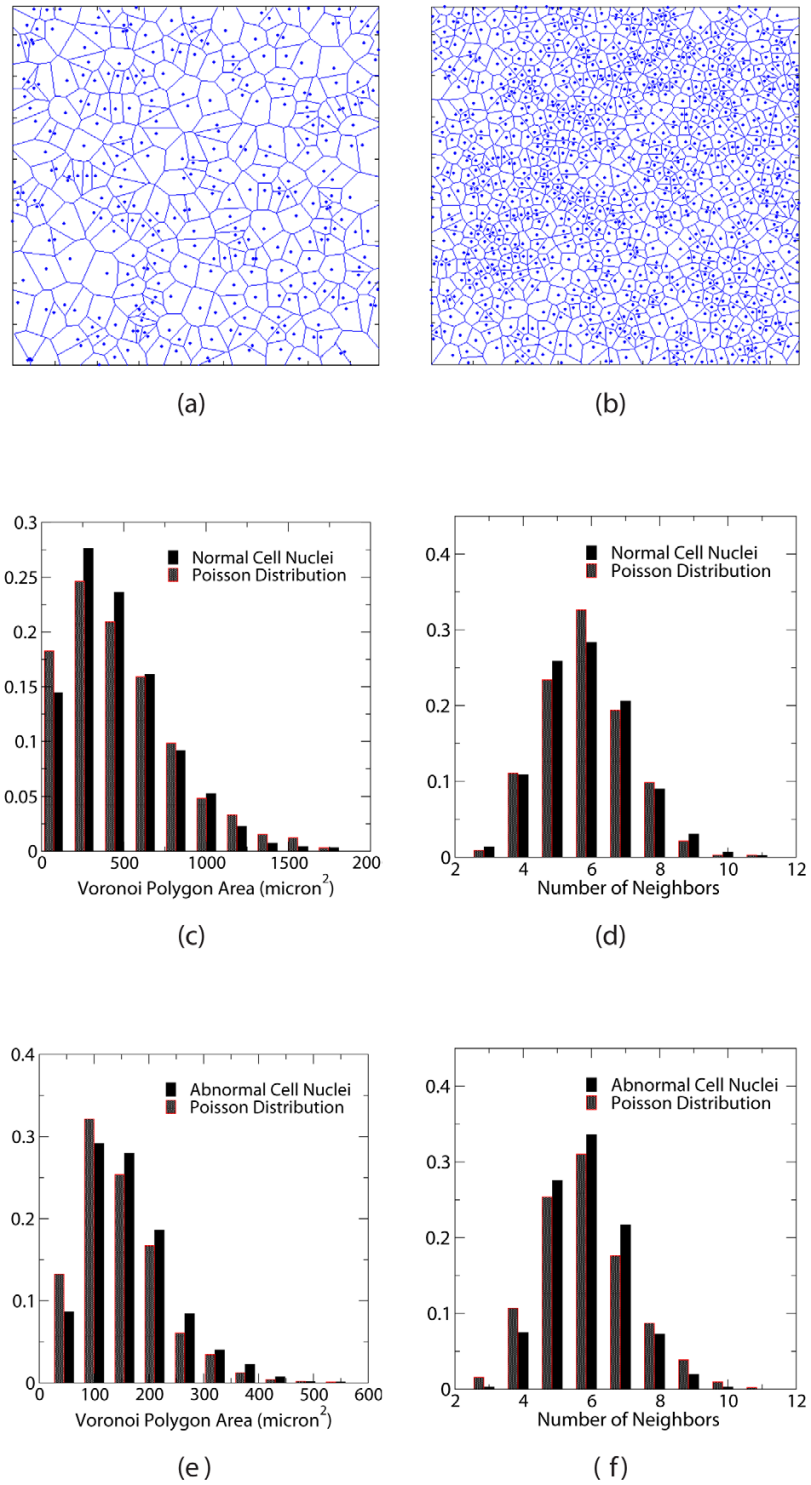
Voronoi statistics (i.e., distributions of Voronoi polygon areas and number of Voronoi neighbors) associated with the distributions cell unclei. Upper panels: A portion of the Voronoi tessellation for normal cell nuclei (a) and abnormal cell nuclei (b). The size of regions shown here is 425 microns by 425 microns. Middle panels: The histogram of the Voronoi polygon areas (c) and the histogram of number of the Voronoi neighbors (d) for normal cell nuclei. Lower panels: The histogram of the Voronoi polygon areas (e) and the histogram of number of the Voronoi neighbors (f) for abnormal cell nuclei.

We first obtain the Voronoi tessellation associated with each point distribution (distribution of cell nuclei) by constructing Voronoi polygons. For a general point distribution in two dimensions, the Voronoi tessellation is a subdivision of the plane into polygons, each of which is associated with a point in the point distribution. Namely, each polygon defines the region of space that is closest to a point than to any other points [8]. The collection of all Voronoi polygons fills the plane without any gaps (see Supporting Information S1 and supporting figure Fig. S2 with the associated legend). Thus, the area of a cell's Voronoi polygon is representative of the space that the cell occupies and cells are considered to be neighbors of one another if their Voronoi polygons share a common edge. The area and the number of nearest neighbors of each Voronoi polygon are then obtained. For each nuclei distribution, such statistics are binned to generate histograms. The histograms are then averaged over different nuclei distributions to produce characteristic Voronoi statistics. Poisson point distributions at corresponding number densities are generated whose Voronoi statistics are also collected and compared to those of distributions of normal and abnormal cell nuclei (see Fig. 2). It can be seen that both normal and abnormal nuclei distributions have a smaller number of Voronoi polygons with smaller areas than that of the corresponding Poisson point distributions, in which two points can get arbitrarily close to each other. This implies that cell nuclei possess an effective repulsion that prevents them getting too close to each other. Except for this distinction, the Voronoi statistics of the nuclei distributions do not significantly deviate from those of corresponding Poisson point distributions. However, as we show in the following sections, the distributions of cell nuclei (especially the nuclei of brain glioma cells) indeed possess nontrivial spatial correlations, which makes them distinctly different from Poisson systems. Therefore, the Voronoi statistics are not able to capture well the salient features of spatial correlations in distributions of the nuclei of either benign brain white matter cells or brain glioma cells.

### Pair Correlation Function and Structure Factor

It is not completely surprising that the Voronoi statistics are not sensitive descriptors, since they are local measures associated with single cells. On the other hand, the pair correlation function 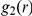 and structure factor 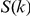, respectively, reflect short-range and long-range spatial correlations in the system at the *two-point level* (see Materials and Methods for details). In other words, 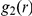 and 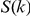 quantify how pairs of cell nuclei are correlated in space and reciprocal space, respectively. Given a point distribution, 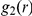 can be easily obtained by computing and binning the separation distances between all point pairs [8], and its value at 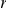 is related to the probability of finding a point at a distance between 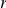 and 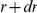 to a reference point in the system. 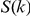 is also computed directly from the distribution of cell nuclei using Eq. (3). For each nuclei distribution, 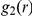 and 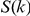 are computed, and the final 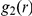
's and 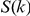
's reported are obtained by averaging over all of the nuclei distributions. We note that salient features of 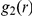 and 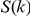 discussed below are observed in each individual distribution, implying that only noisy fluctuations are averaged out.

**Figure 3:**
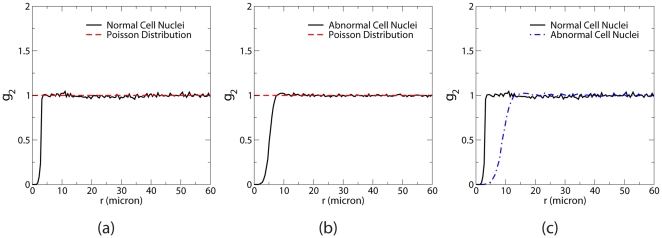
Pair correlation functions associated with the distributions of benign brain white matter and brain glioma cell nuclei. (a) 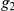 associated with benign brain white matter cell nuclei compared with 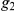 of Poisson point distributions at the same number density. (b) 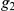 associated with brain glioma cell nuclei compared with 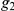 of Poisson point distributions at the same number density. (c) Direct comparison of 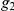 for properly scaled distributions of normal and abnormal cell nuclei.

Figures 3(a) and (b) show 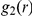 associated with the distributions of the normal and abnormal cell nuclei as well as 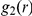 of Poisson distribution of points, which is trivially equal to unity for all values of 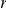, meaning that it is equally likely to find point pairs at all separation distances in such a completely uncorrelated system. For both nuclei distributions, 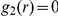 for a range of small-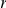 values and rapidly increases to unity, indicating there is an effective repulsion between the nuclei, i.e., no two nuclei can get arbitrarily close to one another in space. Figure 3(c) compares 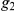 associated with normal and abnormal cell nuclei scaled to the same number density. The slower increase of 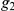 associated with abnormal cell nuclei implies that the effective repulsion between them is stronger than that between the normal cell nuclei, which may arise due to differences in shape and size of normal and abnormal cells.

**Figure 4:**
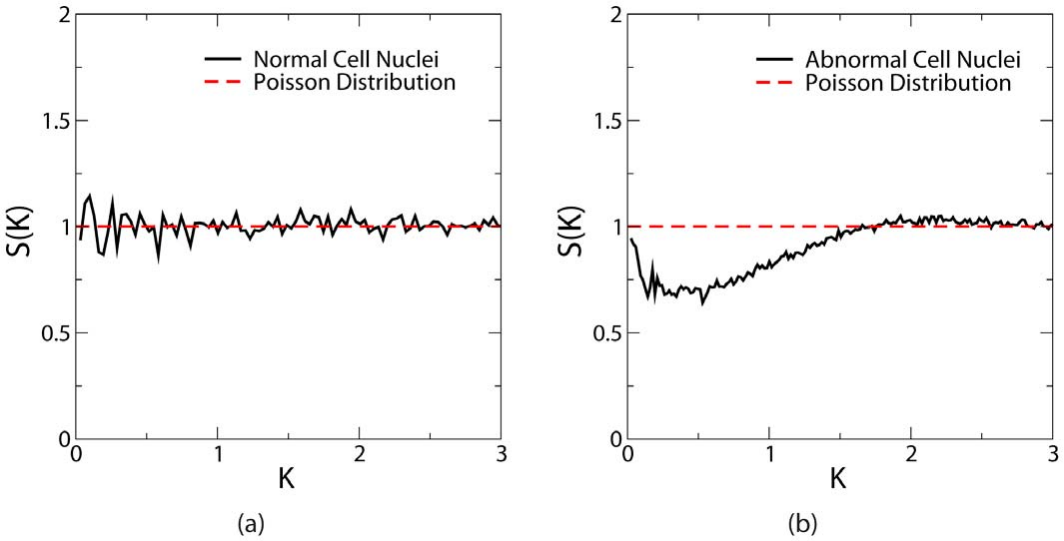
Structure factor 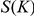 associated with the distributions of benign brain white matter and brain glioma cell nuclei. (a) 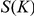 associated with benign brain white matter cell nuclei compared with 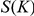 of Poisson point distributions at the same number density. (b) 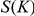 associated with brain glioma cell nuclei compared with 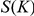 of Poisson point distributions at the same number density. We have used a scaled wavevnumber here 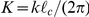, where 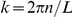 (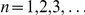, 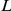 is the linear size the system) is the conventional wavenumber. The characteristic neighbor distances for normal and abnormal cell nuclei are respectively, 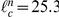 microns and 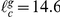 microns. The real-space length scale 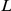 associated with the scaled wavenumber 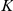 can be obtained via 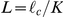.

Figure 4 shows 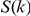 associated with the distributions of normal and abnormal cell nuclei as well as 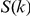 of Poisson distribution of points, which is equal to unity for all values of wavenumber 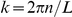 (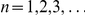 and 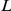 is the linear size the system). Here we use a scaled wavevnumber 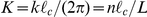, where 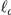 is the characteristic neighbor distance. The real-space length scale 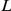 associated with the scaled wavenumber 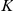 can be easily obtained via 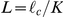. It can be seen that the structure factor associated with the normal cell nuclei does not significantly deviates from unity, implying the lack of long-range spatial correlations between the nuclei. On the other hand, 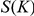 for the abnormal cell nuclei dramatically drops below unity at relatively small wavenumbers and deviates from 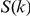 for normal cell nuclei by approximately 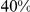. This appreciable dip in the wavenumber range 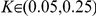 for the abnormal cell nuclei clearly indicates that these systems possess spatial correlations on intermediate and large length scales (i.e., 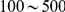 microns), since density fluctuations at these length scales are suppressed [18]. This means that the cell nuclei are organized in a collective way rather than randomly at these large distances. We emphasize that this behavior is observed in 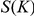 for each individual distribution of abnormal cell nuclei and persists in the averaged structure factor. Such long-range correlations can hardly arise from local packing effects determined by cell shapes and sizes and suggests that there might exist long-range communications between abnormal cells in certain form that would lead to cooperative and collective cell behavior responsible for invasion and metastasis of malignant tumors. The length scale associated with the cell-cell communication could suggest that the correlations are at least in part a function of cellular or non-cellular extra-glial factor(s) (i.e., the tumoral microenvironments). Alternatively, this may be a function of the ultrastructure of networks of glial-cell processes. The significance of this observation will be further addressed in the Discussion.

### Nearest-Neighbor Functions

A direct comparison of 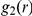 associated with the properly scaled nuclei distributions shows that the effective repulsions between the abnormal cell nuclei are stronger than that between normal cell nuclei. To better understand this effective interaction, we investigate the spatial correlations among *neighboring* nuclei by computing the nearest-neighbor functions, i.e., the “particle” and “void” nearest-neighbor exclusion probability functions 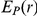, 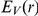, respectively [19], [20], which provide information on the distances between nearest neighboring nuclei and the size of spherical voids in the nuclei distributions (see Materials and Methods).

**Figure 5:**
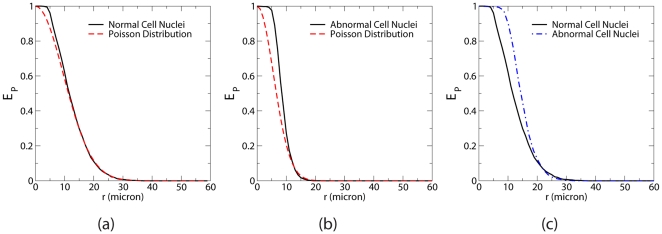
Particle exclusion probabilities 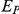 associated with the distributions of benign brain white matter and brain glioma cell nuclei. (a) 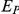 associated with benign brain white matter cell nuclei compared with 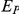 of Poisson point distributions at the same number density. (b) 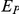 associated with brain glioma cell nuclei compared with 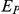 of Poisson point distributions at the same number density. (c) Direct comparison of 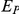 for properly scaled distributions of normal and abnormal cell nuclei.

Figures 5(a) and (b) show 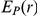 associated with the distributions of the normal and abnormal cell nuclei as well as 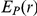 of Poisson point distributions at the same number densities 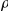, which is 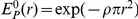 [8]. It can be seen that 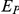 for normal cell nuclei only deviates from (i.e., greater than) that for the corresponding Poisson distribution of points at small 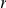 values, indicating an effective short-range repulsion between the neighbor nuclei. On the other hand, 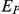 for abnormal cell nuclei significantly deviates from 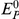 of the corresponding Poisson point distributions for a much wider range of 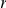 values. In Figure 5(c), we directly compare 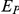 for the properly scaled nuclei distributions. The larger exclusion probabilities associated with the abnormal cell nuclei clearly indicates a stronger repulsion between them, consistent with the conclusions drawn from 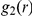 analysis.

**Figure 6:**
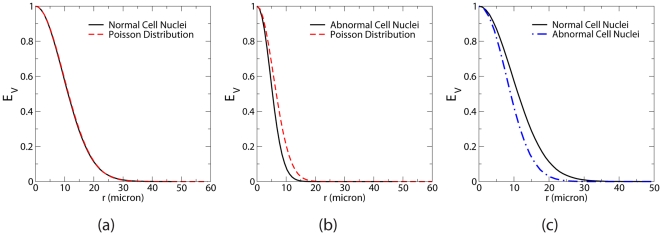
Void exclusion probabilities 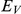 associated with the distributions of benign brain white matter and brain glioma cell nuclei. (a) 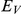 associated with benign brain white matter cell nuclei compared with 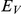 of Poisson point distributions at the same number density. (b) 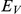 associated with brain glioma cell nuclei compared with 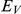 of Poisson point distributions at the same number density. (c) Direct comparison of 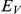 for properly scaled distributions of normal and abnormal cell nuclei.

The void exclusion probability 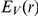 associated with the distributions of the normal and abnormal cell nuclei are shown in Figures 6(a) and (b), together with 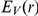 of Poisson point distributions at the same number densities 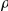, which is 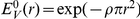 [8]. (Note that for Poisson point distributions, 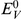 and 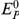 are identical.) We see that the void exclusion probabilities for abnormal cell nuclei are smaller than that for Poisson systems, meaning the voids in such nuclei distributions are smaller due to the stronger nuclei repulsion. (If nuclei could get closer, the voids left behind would be larger in size.) However, 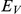 for normal cell nuclei distributions is only slightly below 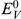 for the corresponding Poisson distribution, which again implies the weaker spatial correlations in these systems. Note that its long (slower decaying) tail indicates the existence of appreciably large voids in the system as the ones found in Poisson point distributions. Same conclusions can be drawn from a direct comparison of 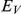 for the properly scaled nuclei distributions shown in Figure 6(c).

Thus, the nearest-neighbor statistics clearly reflect the fact that the effective repulsion between the abnormal cell nuclei is much stronger than that between the normal cell nuclei, which leads to effectively larger distances between nearest neighboring cell nuclei and smaller voids in the distribution. This could cause the reduced extra-cellular diffusion common in many cancers [17].

## Discussion

In this paper, we have characterized the spatial distributions of the nuclei of both benign brain white matter cells and infiltrating glioma cells via a variety of nontrivial statistical microstructural descriptors, including the pair correlation function, structure factor and various nearest neighbor functions that have been profitably utilized in statistical mechanics and material science. To the best of our knowledge, this is the first time that such spatial statistics has been applied in the analysis of histological images. Our primary data was derived from images of clinical microscopic slides and we focused our analysis on cell nuclei because in the CNS, glial and glioma cell borders are not well delineated in routine hematoxylin and eosin stained stained sections. While our GBM images were chosen in areas of high viable tumor cellularity, and histologically apparent non-tumoral structural heterogeneities were subtracted, the minority of non-tumoral nuclei present within our processed GBM images are treated as equal in our analysis. This is a limitation of our study that precludes definitive assignment of the relative contribution of non-malignant cells to the microstructural descriptors we observe as unique to GBM. Addressing this potential limitation requires future identification of molecular markers or other methods that identify malignant cells within GBM with a high degree of specificity (and ideally high sensitivity) across a series of randomly selected GBMs. An alternative approach involves comparative studies following methodical immunohistochemical detection and subtraction of each of the non-malignant cell types within GBM.

We note that GBM masses generally have a small fraction of cells that are multinucleated (i.e., with multiple nuclei in a single cell) [21]. Since such nuclei are confined within single cells, their contributions to the spatial statistics are mainly associated with small-distance values and do not significantly affect the correlations on large length-scales. Although this multinucleation would cause certain discrepancies between the statistics associated with the distributions of cell nuclei and the cells themselves, we expect the discrepancies to be negligibly small on large length-scales. In addition, since the nuclei of the two types of cells appear to be similar in size, we believe that any artificial effects due to sectioning should be small. We also note that small perturbations of the individual nucleus positions do not affect the overall statistics associated with the distributions. Since distributions of both normal and abnormal cell nuclei are statistically homogeneous and isotropic, the conclusions based on the evaluations of the particular correlation functions of the 2D histological images examined in this paper should also apply in 3D nuclei distributions [8]. Although the 3D Voronoi statistics will be quantitatively different than those in 2D, in terms of the deficiency of not being able to capture long-range correlations, our conclusion also holds.

For comparison purposes, we have also investigated the Voronoi statistics associated with the nuclei distributions. We have demonstrated that while Voronoi statistics cannot clearly distinguish structural differences between normal and abnormal cell nuclei beyond length scale associated with single cells, their salient structural distinctions are very well captured by the aforementioned correlation functions. In particular, by comparing the statistics of the nuclei distributions to the corresponding Poisson reference systems and by directly comparing properly scaled distributions of the nuclei, we have shown that there exist effective repulsions between both normal and abnormal cell nuclei; and that the repulsions between the abnormal cell nuclei are much stronger than that between the normal cell nuclei. This repulsion could simply result from exclusion-volume effects of the cytoplasm (i.e., one cell cannot occupy the same space as another cell) or it could be caused by the competition between local cells for nutritional needs. In addition, abnormal cell nuclei pack considerably more densely and are more spatially correlated than the normal cell nuclei, which is not completely surprising given the corresponding differences in their proliferation rates, nutritional needs and motilities. This in turn leads to deviations between their correlation functions at small length scales (i.e., the characteristic neighbor distances).

Importantly, we found that abnormal cell nuclei possess nontrivial spatial correlations on intermediate and large length scales, as manifested by the strong suppression of cell-density fluctuations on these length scales. This observation is revealing and appears to be new and biologically significant. Such long-range correlations can hardly arise from local packing effects determined by cell shapes and sizes. Possible mechanisms for these long-range correlations include altered structural or cellular components of the tumoral microenvironments. For example, subpopulations of glioblastoma cells can organize around a vascular niche [22]. Alternatively, as glial cells are known to generate complex networks of cellular processes [23], the spatial correlations may be maintained by the ultrastructure of glial-derived processes. These possibilities enable a “mutualism” mechanism in which abnormal cells can survive in the stressful tumor environment based on “common goods” principles. There is increasing evidence that cooperative and collective cell behavior plays an important role in the invasion and metastasis of malignant tumors. The observed long-range spatial correlations between abnormal cell nuclei clearly supports the view that tumors are complex dynamic and self-organizing systems rather than a random (unorganized) collection of cells.

This work also provides the structural characteristics of brain glioma cells and sensitive statistical descriptors, which can have potential applications in cancer diagnosis. Recently, analysis of the alterations in nuclear structure [24] and wavelet methods [25] have been employed to analyze histological samples of prostate cancer and the obtained statistics can be used to devise a classification scheme of the malignancy of the tumor. Our analysis suggests that characterizing distributions of cell nuclei via correlation functions provides a complementary way to analyze histological samples and may have utility in advancing the development of computer assisted diagnostic pathology technologies. The unique patterns of cell nuclei distributions may be a measurable bio-marker of tumor behavior and tumor phenotypes over larger length scales and therefore, may have applications in assessing the extent of infiltration and margin status from a limited sample.

Finally, we note that the specific correlation functions employed here are just a small subset of the zoology of known sophisticated statistically descriptors, including those that have recently been fruitfully applied to characterize the microstructure of heterogeneous media [26]. Our studies lay the groundwork for future biological investigations that seek to quantify the relative roles of tumors cells and non-neoplastic cells in shaping the organization of tumoral microenvironments via the descriptors reported here or even more sophisticated correlation functions. The ability to assay the collective behavior of cancer cells provides new opportunities to impede malignant progression through the targeting of tumor self-organization. Moreover, these microstructural descriptors may also have fruitful applications in the study of morphogenesis, for which understanding the spatial correlations among cells is crucial.

## Materials and Methods

In this section, we briefly describe how the histological images are obtained and define the statistical descriptors that we employed to characterize the spatial distributions of cell nuclei, which include the pair correlation function 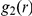, structure factor 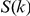 and nearest neighbor functions 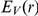 and 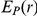. In statistical mechanics and material sciences, these functions have been used to provide indispensable structural information of systems composed of interacting particles. Here, we consider the cells as “particles” whose geometrical centroids coincide with the centroids of their nuclei. These correlation functions provide quantitative information on how the spatial arrangement of a cell is affected by the presence of another cell a prescribed distance away. We note that this study received research ethics board approval at Princeton University and the University of Toronto. Informed consent from all participants involved in the study was obtained in written form.

### Obtaining Histological Images

Light microscopic images from H&E stained sections were obtained on a Leica DM4500B microscope/DFC420 camera/10X/0.40 objective (total magnification 246X) with Leica Application Suite v3.7.0 and captured format settings of 2592×1944 interlaced large HQ. Final images were stored and analyzed in 300 dpi JPEG format.

### Pair Correlation Function and Structure Factor

It is well known in statistical mechanics that a classical many-particle system can be completely characterized by a countably infinite set of probability density functions associated with finding a particular distribution of particle centers in space, i.e., 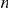 *-particle correlation functions*. The 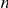-particle correlation function 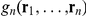 is proportional to the probability density of finding 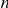 particles in differential volume elements around the positions 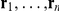, regardless of the positions of the remaining particles in the system. For a *statistically homogeneous* system, 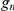 is translational invariant and hence depends only on the relative displacements of the positions with respect to some chosen origin, say 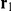: 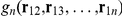, where 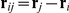. For an arbitrary system, deviations of 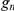 from unity provide a measure of the correlations among the particles in the system.

Of particular interest is the pair correlation function 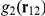. When the system is also *statistically isotropic*, 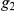 depends on the radial distance 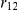 only, i.e., 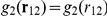, which defines the average number of particle centers surrounding a reference particle center. In particular, 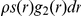 is proportional to the conditional probability of finding a particle center in the spherical shell of volume 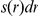 (where 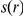 the surface area of the sphere shell with radius 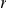), and 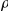 is number density of the system.

In this paper, 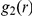 is employed to characterize spatial correlations between pairs of cell nuclei on relatively small length scales. Since two cells can never occupy the same space due to cytoplasm exclusion effects (described earlier), the probability of finding two cells at the same place (i.e., 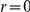) is identically zero, i.e., 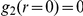. At finite separations, the position of one cell is generally influenced by the other through various intercellular biomechanical/biochemical signals, which leads to variations in the probability of finding cell nuclei at certain distances away from a reference nuclei.

For statistically homogeneous and isotropic systems, the structure factor 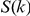 is defined as follows:
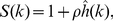
(1)where 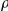 is the number density, 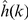 denotes the Fourier transform the total correlation function 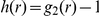 and the wavenumber 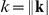 is the magnitude of the reciprocal variable to 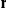. We utilize the following definition of the Fourier transform:
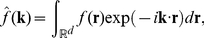
(2)where 
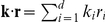
 is the conventional 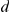-dimensional inner product of two real-valued vectors in 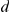-dimensional Euclidean space 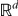.

For disordered systems, the small 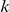 behavior of 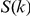 reflects the long-range correlations in the system in real space. Moreover, the small-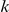 behavior is related to the manner in which 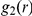 approaches its large-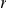 asymptotic value of unity, *not* the asymptotic value itself. However, it is well known that it is extremely difficult to accurately capture the large-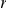 behavior of 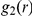 by direct sampling. Thus, 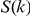 is not computed using Eq. (1), but rather is obtained directly from the distribution of the particle centers in the system as encoded in the collective coordinate density 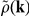, i.e.,
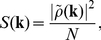
(3)where 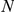 is the number of particles in the system and 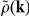 are defined as
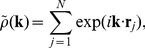
(4)where 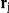 denotes the location of particle 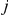. We note that the wavevector 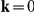 (associated with the forward scattering density) should be excluded when computing the structure factor using Eq. (3). Recall that we have employed periodic boundary conditions, which leads to discrete values of the wavevector and the associated angularly averaged wavenumber 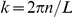, where 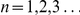 and 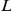 is the linear size of the system.

In other words, 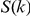 for small 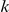 values reflects the degree to which there exists large-scale collective organizations in the spatial distributions of the cell nuclei. If the cells are not correlated on large length scales, e.g., the position of a cell is not affected by another cell far away, the structure factor is equal to unity for all values of 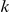. If the distribution of cells possess long-ranged spatial correlations, variations in 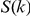 from unity should be observed.

### Nearest-Neighbor Functions

In considering a system of interacting particles, it is important to understand the effects of the nearest neighbor on some reference particle in the system. This requires knowledge of the probability associated with finding the nearest neighbor at some given distance from a reference particle, i.e., the *particle* exclusion probability function 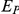 [19], [20]. A different nearest-neighbor function 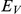, which is a more fundamental quantity [27], characterizes the probability of finding a nearest-neighbor particle center at a given distance from an *arbitrary* point in the system [19] and is referred to as the *void* exclusion probability function.

Formally, 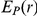 and 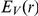 are defined as follows:
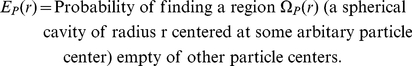
(5)

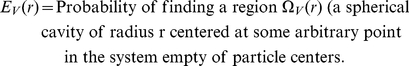
(6)Note that the void exclusion probability 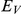 can also be interpreted as the expected fraction of space available to a “test” sphere of radius 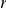 inserted into the system, and thus, provides nontrivial void information of the system. Both 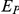 and 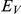 are monotonically decreasing functions of 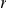 [8].

The nearest-neighbor functions 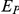 and 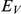 reflect how cells are *locally* arranged with respect to their immediate neighbors. At high cellular densities, the spatial arrangement of neighboring cells is largely determined by the cytoplasm exclusion volume effects. Specifically, the positions of the neighboring cells are expected to be more correlated so that they can occupy the available space more efficiently (i.e., pack more densely). At low cellular densities, the exclusion volume effects are weaker and the spatial arrangement of neighboring cells could be less correlated.

## Supporting Information

Figure S1 **Point configurations with various degrees of spatial correlation.** (a) A Poisson distribution of points generated by randomly placing a large number of points in a square box. The points are spatially uncorrelated and two points can get arbitrarily close to one another. (b) A point configuration associated with the random sequential addition (RSA) of nonoverlapping circular disks. Disks are sequentially and randomly added subject to the nonoverlapping constraints. The points correspond to the centers of the disks. Note that this configuration is more spatially correlated than the Poisson distribution of points as explained in the text. (c) Points on the sites of the triangular lattice. The points are completely correlated with one another.
(EPS)

Figure S2 **Voronoi tessellations associated with two-dimensional point configurations shown in** **Fig. 1** **.** (a) Voronoi tessellation of a Poisson point configuration. (b) Voronoi tessellation of RSA disk centers. (c) Voronoi tessellation of triangular-lattice point configuration.
(EPS)

Supporting Information S1 (PDF)
