# Supplementary material for: Spatial Organization and Correlations of Cell Nuclei in Brain Tumors
Source: PLoS One. 2011 Nov 16;6(11):e27323. doi: 10.1371/journal.pone.0027323 (PMC3217938; doi:10.1371/journal.pone.0027323)
Supplement: Supporting Information S1 — (PDF) [file pone.0027323.s003.pdf]

Supporting Information: Spatial Organization and Correlations of Cell Nuclei in Brain Tumors

Point Configurations with Various Degrees of Spatial Correlations

In the main paper, we used Poisson distribution of points as reference system, whose spatial statistics are compared to those associated with point configurations mapped from distributions of cell nuclei. A Poisson point configuration can be constructed by randomly placing a prescribed number of points in a domain with fix volume, regardless the positions of existing points. Therefore, the points are completely uncorrelated to each other in a Poisson distribution.

In general, point configurations (i.e., distributions of points) with various degrees of spatial correlations can be constructed. Figure 1 of this text shows three examples in two dimensions: a Poisson point configuration, the centers of a packing of nonoverlapping equal-sized disks obtained by the random sequential addition (RSA) process, and the points arranged on the sites of the triangular lattice. In the distribution of RSA disk centers, no disk can overlap any other disks (i.e., the distance between any two disk centers must not be smaller than the diameter of the disks), which imposes spatial correlations between the centers. In the triangular lattice of points, the positions of any two points are rigorously related by a linear combination of two basis vectors for the lattice. Therefore, all of the points in the triangular lattice are completely correlated with each other.

Distributions of points with different degrees of spatial correlations generally possess spatial statistics (e.g., the pair-correlation function g_2 as defined in the main paper) that deviate from those of a Poisson distribution. Such statistics can provide a measure of spatial correlations in the system, as we have shown in the main paper.

Voronoi Tessellations Associated with Various Two-Dimensional Point Configurations

Every point configuration can be associated with a Voronoi tessellation, which is a fundamental tiling of space into polyhedra (see the main text). The polyhedra are referred to as Voronoi cells, which are the regions of space nearer to their associated points than to any other points in the configuration.

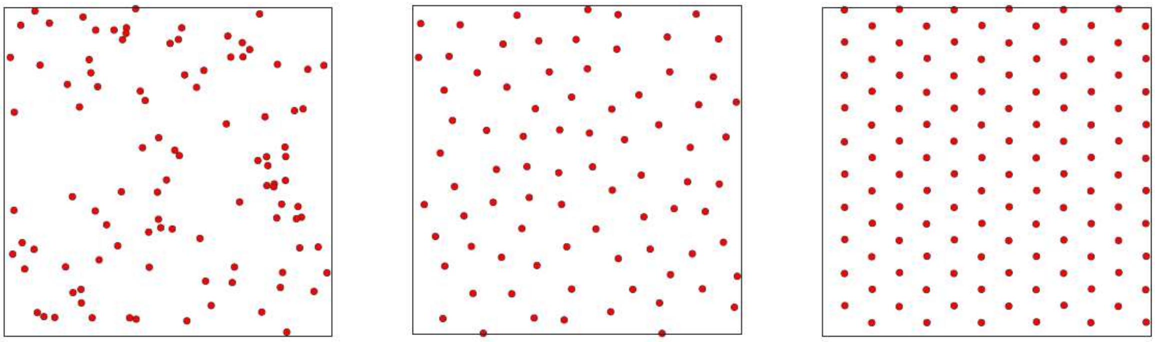

Figure 1: Point configurations with various degrees of spatial correlation. (a) A Poisson distribution of points generated by randomly placing a fixed number of points in a square box. Note that the points have no spatial correlations and two points can get arbitrarily close to one another. (b) A point configuration associated with the random sequential addition (RSA) of nonoverlapping circular disks. The points correspond to the centers of the disks. Note that this configuration is more spatially correlated than the Poisson distribution of points as explained in the text. (c) Points on the sites of the triangular lattice. The points are completely correlated with each other.

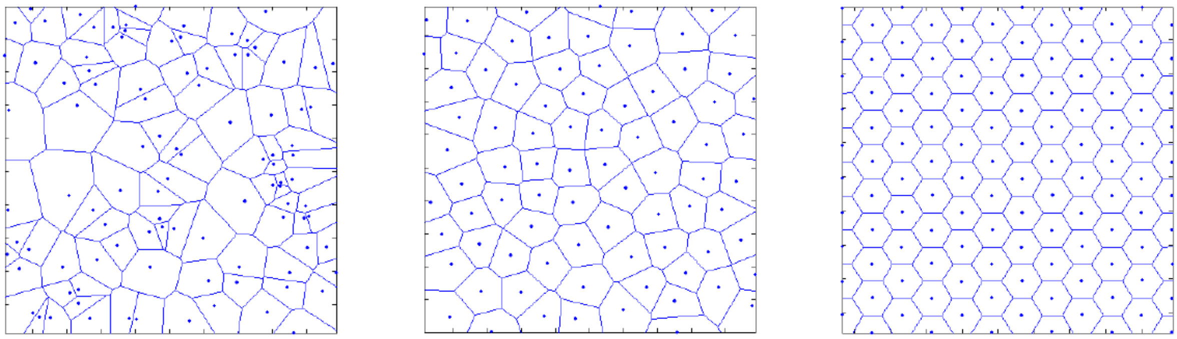

Figure 2: Voronoi tessellations associated with two-dimensional point configurations shown in Fig. 1. (a) Voronoi tessellation of a Poisson point configuration. (b) Voronoi tessellation of the RSA disk centers. (c) Voronoi tessellation of the triangular-lattice point configuration.

In the main paper, we have shown the Voronoi tessellations associated with the distributions of cell nuclei. In Figure 2 of this text, we show the Voronoi tessellations of various point configurations in two dimensions, including the Poisson distribution of points, the centers of a packing of nonoverlapping equal-sized disks obtained by the random sequential addition (RSA) process, and the points arranged on the sites of the triangular lattice (see Fig. 1 of this text). Although the Voronoi tessellations associated with these point configurations are distinguishable, they are not very efficient spatial characteristics of the distributions of cell nuclei in healthy and tumorous environments, as we have shown in the main paper.
